# Interaction between saliva’s adenosine and tick parasitism: effects on feeding and reproduction

**DOI:** 10.1186/s13071-017-2248-8

**Published:** 2017-07-10

**Authors:** Elen Anatriello, Carlo José Freire Oliveira, Nathália Baptista Oliveira, Andressa Fisch, Cristiane Maria Milanezi, João Santana da Silva, Isabel Kinney Ferreira de Miranda-Santos, Beatriz Rossetti Ferreira

**Affiliations:** 1 Institute of Science and Technology, Federal University of São Paulo, UNIFESP, Rua Talim, 330, São José dos Campos, São Paulo, 12231-280 Brazil; 2 Institute of Biological and Natural Sciences, Federal University of Triângulo Mineiro, UFTM, Praça Manoel Terra, 330, Uberaba, Minas Gerais 38015-050 Brazil; 3 Department of Maternal and Child and Public Health Nursing, Ribeirão Preto School of Nursing, University of São Paulo, USP, Avenida Bandeirantes, 3900 Ribeirão Preto, São Paulo, 14040-902 Brazil; 4 Department of Biochemistry and Immunology, Ribeirão Preto School of Medicine, University of São Paulo, USP, Avenida Bandeirantes, 3900 Ribeirão Preto, São Paulo, 14049-900 Brazil

**Keywords:** Ticks, *Rhipicephalus sanguineus*, Saliva, Adenosine, Dendritic cells, T cells

## Abstract

**Background:**

It has recently been demonstrated that saliva from *Rhipicephalus sanguineus* ticks contains adenosine (ADO) and prostaglandin E2 (PGE2), two non-protein molecules that have significant immunomodulatory properties. These molecules can inhibit cytokine production by dendritic cells (DCs), while also reducing the expression of CD40 in these cells. However, more studies are needed for a better understanding of their participation in the feeding of ticks in vivo. This work, therefore, evaluated the importance of ADO during tick infestations. Mice were infested with adult ticks (3 couples/mouse), and their skin was collected at the tick-infested site (3rd and 7th day), and mRNA for receptors of ADO was quantified by real-time PCR.

**Results:**

Tick infestation increased by four and two times the expression of the A2b and A3v1 receptors on day 3, respectively, while expression of other ADO receptors was unaltered. In addition, we treated mice (*n* = 10/group) daily with 8-(p-Sulfophenyl)theophylline, 8-pSPT, 20 mg/kg, i.p.), a non-selective antagonist of ADO receptors, and evaluated the performance of ticks during infestations. Female ticks fed on 8-pSPT-treated mice presented a reduction in their engorgement, weight and hatching rates of egg masses, and survival times of larvae compared to the same parameters presented by ticks in the control group. To investigate if these 8-pSPT-treated mice presented altered immune responses, we performed three tick infestations and collected their lymph node cells to determine the percentages and activation state of DCs and cytokine production by lymphocytes by flow cytometry (Cytometric Bead Array technique, CBA). Our data showed that 8-pSPT-treated mice presented an increase in the percentage of DCs as well as of their stimulatory and co-stimulatory molecules (CD40, CD80 and MHCII). Regarding production of T cell cytokines, we observed a significant increase in the levels of IL-2 and a significant decrease in IL-10, IL-17, TNF-α and IFN-γ cytokines.

**Conclusions:**

These results suggest that ADO produced by ticks helps them feed and reproduce and that this effect may be due to modulation of host DCs and T cells.

## Background

Over the past few years, the brown dog tick (*Rhipicephalus sanguineus*) has taken a very important role among the species of ticks in Brazil and worldwide [1–3]. This is due to the direct effects of parasitism and the transmission of disease-causing agents to both dogs and humans [4–8].

The *R. sanguineus*-host interaction at the skin feeding site is complex. Thus, to succeed during the parasitic phase, these ticks need to overcome the barriers imposed by the host response [9–12]. The first barrier is represented by the hemostatic system, while the second one is imposed by immune cells and molecules of innate and acquired immune response. The hemostasis and immune response appear during the tick attachment phase when the arthropod breaks the physical barrier of the host (skin) and inoculates its saliva to facilitate their access to blood, as well as escape from other host defences systems [11, 13].

The number and variety of anti-hemostatic and immunomodulatory components present in tick saliva are so vast that this parasite has been considered a “professional pharmacologist” and has been studied as a source of numerous therapeutic targets [11, 14–16]. The anti-hemostatic and immunomodulatory activities of tick saliva [11–13, 17] include the modulation of vasodilation [17, 18], inhibition of platelet aggregation [19], reduction of T lymphocyte proliferation and function [20, 21], suppression of the activity and production of cytokines and chemokines [21–23], inhibition of maturation and function of macrophages [21, 23, 24], dendritic cells (DCs) [25–29], neutrophils [30], mast cells and natural killer (NK) cells [24], and containment of mediators essential for rash and pain induction [31].

Given the induction of innate and acquired immune response is triggered mainly by DCs, since they are specialised in activating naïve lymphocytes and initiating primary immune responses [32], our group has investigated, whether the modulation of immune response mediated by tick infestation is dependent on these cells. We have shown that the saliva of *R. sanguineus* ticks inhibits DCs differentiation, reduces the expression of stimulatory and co-stimulatory molecules like CD40, CD80 and CD86, and modulate DCs cytokine production [26–28]. Moreover, we showed that its effect, at least in vitro, is dependent in part on adenosine (ADO) and prostaglandin E2 (PGE2), two non-protein molecules contained in the saliva that have multiple immunomodulatory properties [29].

ADO is an endogenous purine nucleoside that modulates a wide variety of functions in several cells of the immune system, including DCs, T and B cells, within others [33–36]. In order to exert its activity, ADO binds specifically to a family of four G-protein receptors, named A1, A2a, A2b, and A3 receptors. Interestingly, ADO exercises its anti-inflammatory/ immunosuppressive effects by binding to the A2a and A2b receptors, whereas the binding to the A1 and A3 receptors results in pro-inflammatory actions [37–39].

Importantly, ADO has been identified in salivary glands of other classes of hematophagous arthropods, notably, the sand flies *Phlebotomus argentipes* and *Phlebotomus duboscqui* [40, 41]. In addition, it has been shown that ADO and AMP in *Phlebotomus papatasi* saliva mediate the exacerbating effects of *Leishmania* infection by promoting a tolerogenic profile in DCs and by differentiating inducible T regulatory cells in the inflammatory site through an A2a receptor mechanism [42].

The present study evaluated, for the first time, the in vivo participation of ADO during infestation of mice by *R. sanguineus* ticks. We studied the expression of ADO receptors, the feeding and reproductive parameters of ticks, and the immune response of tick-infested mice treated or not with a non-selective antagonist of ADO receptors. Moreover, we tested if knockout (KO) mice for A2a receptors were enhanced in their resistance to ticks. The study of the role of ADO in a tick infestation can contribute to a better understanding of the tick-host interface.

## Methods

### Colony of ticks and infestation

*Rhipicephalus sanguineus* ticks were laboratory-reared as previously described [43]. For tick infestation experiments, BALB/c mice (10 animals per group) were infested with three pairs of adult *R. sanguineus* ticks restricted in plastic feeding chambers fixed to their backs, as previously described [21]. BALB/c mice were three times tick-infested (7–15 days/per infestation), with an interval of 30 days between each infestation, and animals were treated daily with a non-selective antagonist of ADO receptors (8-pSPT, 20 mg/kg i.p.) or with saline (see Table 1 for the experimental design). During the successive infestations (always on different skin sites), the ticks were evaluated for their biological parameters, i.e. the average weight of the engorged females, egg mass weight, reproductive index, larva hatching rate and larva survival period. In all the experiments of infestation we added a group of Sham animals, a control group consisted of mice that had the chamber fixed to their backs, but had no ticks released. This group was relevant to avoid misinterpretations due to the effect of the glue used to fix the chambers.

**Table 1:** Experimental design

Analysed parameters	Groups			
	First infestation on WT mice	First and second infestation +8-pSPT-treated WT mice	Third infestation +8-pSPT-treated WT mice	First and second infestation on A2aR−/− mice
Expression of the ADO and PGE2 receptors	−	−	−	−
Performance of ticks	−	+	−	+
Expression of stimulatory and co-stimulatory molecules	−	−	+	−
Cytokine production	−	−	+	−

+, Done; −, Not done; 8-pSPT, non-specific antagonist for ADO receptors

### Mice

BALB/c and KO mice for A2a receptors (A2Ar−/− mice on a BALB/c background) [44] 6-week USA) and bred/maintained at the Central Animal Facility of the School of Medicine-USP, Ribeirão Preto. These animals were housed in temperature-controlled rooms (22–25 °C) and received water and food *ad libitum*.

### Real-time polymerase chain reaction for ADO receptors (A1, A2a, A2b and A3v1 and A3v2) and PGE2 receptors (EP1, EP2, EP3 and EP4)

Skin samples from mice one-time tick-infested (3 and 7 days tick-infested) or non-infested (Sham animals; control group) were collected from the area where the ticks were attached or the corresponding site on the Sham mice and placed into a metal block (Biospec, Bartlesville, OK, EUA), added to liquid nitrogen and pulverized using a hammer.

The total RNA was extracted using the TRIzol reagent (Invitrogen, Carlsbad, CA, USA) and the SV Total RNA Isolation System Kit (Promega, Madison, WI, USA), according to the manufacturer’s instructions. Complementary DNA was synthesised using ImProm-II TM Reverse Transcription System (Promega, Madison, WI, USA). SYBR Green Mix-based quantitative real-time PCR assays were performed using the StepOnePlus Real-Time PCR System (Applied Biosystems, Singapore, Malaysia). The mean threshold cycle (Ct) values of measurements were used to calculate the expression of the target gene, which was normalised to the housekeeping gene β-actin, using the 2^-ΔΔCt^ formula.

The primers were the following: A1: 5′-GTT GCC AGC AGT TTT GCC CAC TC-3′, 5′-AGC CCG CAG GGG CTC ATA TCA-3′, A2a: 5′-TTC TTC GCC TGC TTT GTC CT-3′, 5′-ATA CCC GTC ACC AAG CCA TT-3′, A2b: 5′-CTG CTC ATA ATG CTG GTG ATC T-3′, 5′-ATC AGT TCC ATG CGC TGA-3′, A3v1 (isoform 1): 5′-CTA CGC CTG CAA AAT AAA AAA G-3′, 5′-GTC CAA AGA ATC TGA GGT CTG A-3′, A3v2: isoform 2): 5′-CAA AAG CAT CAG TAG AAA CCC A-3′, 5′-ACC GCA CTT CAA ATC CTT GCC-3′, β-actin: 5′-CCT TCC TTC TTG GGT ATG GAA T-3′, 5′-TGG CAT AGA GGT CTT TAC GGA T-3′, EP1: 5′-GTT GCC AGC AGT TTT GCC CAC TC-3′, 5′-AGC CCG CAG GGG CTC ATA TCA-3′, EP2: 5′-GGC CGG AAG GGA GCT CTG GA-3′, 5′-CGG AGG GTC TGA TGG CCC CA-3′, EP3: 5′-GCT ATC CCG CAG CTG AGC CG-3′, 5′-GGG AAA GGC CAC GGA CAC GG-3′, EP4: 5′-GGT CCT GAA CAT CTG AGG CCT GAG C-3′, 5′-CGC GTT GAC TCC GGG GAT GGA-3′.

### Evaluation of biological and reproductive parameters of ticks feeding on mice treated or not with an ADO receptor antagonist or KO mice for A2a receptors

To investigate the in vivo participation of ADO during an infestation of mice by *R. sanguineus* ticks, we treated mice with an antagonist of ADO receptors and infested mice deficient for A2a receptors.

For the assay with the ADO receptors antagonist, BALB/c mice were infested three times (interval of 30 days between infestations) with three couples of *R. sanguineus* adult ticks and treated daily with a non-selective antagonist of ADO receptors (8-pSPT, 20 mg/kg/100 μl i.p.) or saline (*n* = 10 per group) throughout all infestations (9–13 days/infestation). After tick detachment, the biological and reproductive parameters were measured (average weight of the engorged females, egg mass weight, reproductive index, larva hatching rate and larva survival period). The reproductive index was calculated by dividing the egg mass weight (g)/female engorged weight (g) × 100. The biological and reproductive parameters of the ticks were only measured on the first and the second infestations since the mice infested three times were killed and used to evaluate DCs activation and cytokine production on the third or seventh day of the third infestation.

To test if KO mice for A2a receptors (A2aR−/−) would present enhanced resistance to ticks, BALB/c A2aR−/− mice and wild-type mice (WT) (*n* = 10 per group) were one-time tick-infested with three couples of *R. sanguineus* adult ticks and evaluated for similar biological and reproductive parameters as described above, added to the determination of the engorged female number detached for each group.

### Evaluation of DCs activation and cytokine production after treatment with ADO receptor antagonist in mice infested with ticks

BALB/c mice were infested three times (interval of 30 days between the infestations) with three pairs of *R. sanguineus* ticks. These animals were treated daily with 8-pSPT (20 mg/kg, i.p.) or saline. Mice were killed on the third and seventh day (3D and 7D) of the third infestation and cells from lymph nodes draining the infestation site (axillary and brachial lymph nodes) were cultured with and without Concanavalin A (Con-A; 2 mg/mL). After 24 h of culture at 37 °C in 5% CO_2_, the supernatants were collected and stored at -80 °C until use. To study DCs activation, the cells were labelled with monoclonal antibodies conjugated with fluorochromes against CD11c, CD80, CD86, CD40 and MHCII (I-A/I-E) molecules (Biolegend, San Diego, CA, USA). Briefly, after cells were fixed in PBS plus 2% formaldehyde, they were incubated for 30 min with the specific antibodies. Subsequently, the cells were washed with a permeabilization buffer (PBS added fetal bovine serum 1%, sodium azide 0.1% and saponin 0.2%), followed by washing with PBS and re-suspension in 100 μl of PBS. Fluorescence was measured on an FACSCanto Reading II (Becton Dickinson, San Jose CA, USA) obtained in 100,000 events/sample and analysis were performed using the Flow Jo software (TreeStar, Ashland, OR, USA). DCs were firstly gated based on their characteristic of size (FSC) and granularity (SSC), and secondly for the expression of CD11c, MHCII, CD40, CD80 and CD86 markers.

The cytokine levels in the supernatants were determined by Cytometric Bead Array (CBA technique) using the mouse Th1/Th2/Th17 CBA Kit following the manufacturer’s instructions (BD Biosciences, San Jose, CA, USA). Cytokine analysis was performed on the BD™ FACSCanto Reading II (BD Biosciences). All the quantitative analysis was done using FCAP Array™ Software (BD Biosciences).

### Statistical analysis

Data were analysed using GraphPad PRISM 5.0 software (GraphPad Software Inc., La Jolla, CA) and presented as mean ± SEM. Comparisons between animal groups were performed using unpaired Student *t*-test, or the Mann-Whitney test for samples with nonparametric distributions.

## Results

### Tick infestation increased mRNA expression of the ADO receptors A2b and A3v1 in mouse skin

**Fig. 1:**
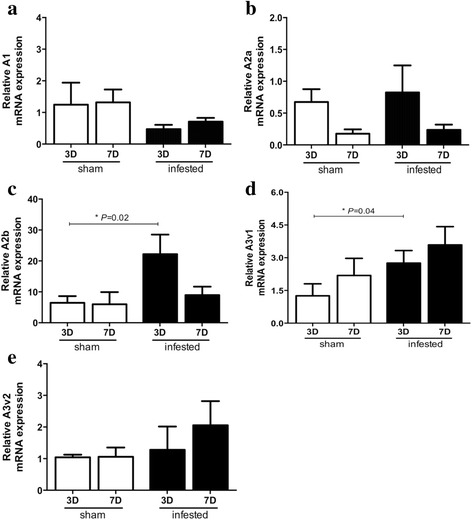
The relative mRNA expression of the ADO receptors in mice skin. Skin samples from one-time tick-infested and sham BALB/c mice; control group (5 to 9/group) were collected on the 3rd and 7th day (3D and 7D) after infestation and processed for assessment of gene expression of receptors for adenosine by real-time PCR. Data are presented as mean values ± SEM of relative expression of A1 (**a**), A2a (**b**), A2b (**c**), A3v1 (**d**) and A3v2 (**e**) mRNA for a target gene normalized for RNA expression of the housekeeping β-actin gene

The results showed that the tick infestation (just one infestation) induced a four and a two-fold increase in the mRNA expression of A2b and A3v1 receptors, respectively on day 3, while no other ADO receptors were significantly altered when compared with the Sham control; control group (Fig. 1). In addition, on the 7th day of infestation, both groups responded similarly. We also investigated the expression of mRNA for PGE_2_ receptors (EP2, EP3 and EP4), and the results showed that the skin of mice infested with ticks presented unchanged mRNA expression for PGE_2_ receptors at the time points analysed (Table 2).

**Table 2:** The relative mRNA expression of the PGE2 receptors in one-time tick-infested and non-infested (sham group; control group) mice skin

	Expression of the target gene normalised to the housekeeping gene β-actin using the 2^-ΔΔCt^ formula		
Groups	EP2	EP3	EP4
3D control	1.543 ± 1.175	0.843 ± 0.475	1.001 ± 0.042
3D infested	0.395 ± 0.195	0.634 ± 0.463	0.432 ± 0.186
*P*-value	0.29	0.78	0.09
7D control	1.054 ± 0.219	1.003 ± 0.055	1.623 ± 1.103
7D infested	0.693 ± 0.183	0.749 ± 0.281	1.766 ± 0,665
*P*-value	0.26	0.48	0.91

*N* = 3–5/group; D, days

### Treatment of mice with an ADO receptor antagonist reduced tick-feeding performance on the first infestation

We treated mice daily with 8-pSPT, a non-selective antagonist of ADO receptors, during the first and second infestation to evaluate the performance of ticks during an infestation when ADO signalling is blocked.

**Fig. 2:**
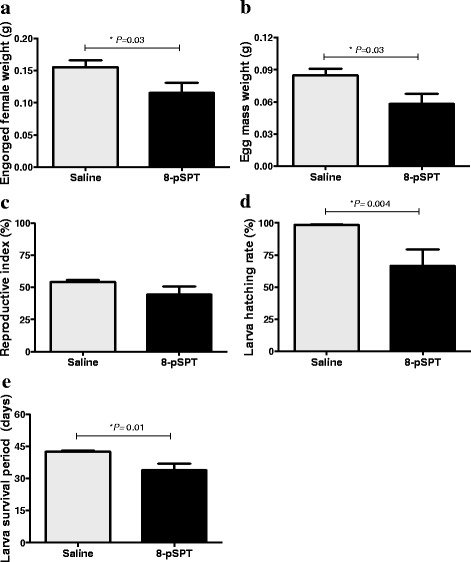
Performance of ticks feeding on mice treated with an ADO receptor antagonist. BALB/c mice (*n* = 10/group) were infested with three couples of adult ticks and treated daily with a non-selective antagonist of ADO (8-pSPT, 20 mg/kg i.p.) or saline. After tick detachment, female ticks were weighed (**a**) and placed in a chamber for oviposition. The egg mass weight (**b**) was measured 15 days after the engorged female tick detachment and after the reproductive index (**c**) larva hatching rate (**d**) and larva survival period (**e**) was calculated. Data are presented as mean values ± SEM from two independent experiments

In the first infestation, we observed that ticks fed on 8-pSPT-treated mice presented a significant reduction in several biological parameters analysed; i.e. engorgement weight of females, egg mass, hatching rate and the larva survival period (*P* < 0.05). The engorgement weight of females showed an average of 0.155 g for ticks fed on the control mice, with a variation ranging from 0.115 to 0.186 g, while for the 8-pSPT-treated mice the mean weight was 0.115 g, with a range from 0.017 to 0.156 g (Mann-Whitney U-test: U_(8)_= 12, *P* = 0.03, Fig. 2a). For the egg mass, the mean value was 0.084 g (ranging from 0.053 to 0.104 g) and 0.058 g (ranging from 0 to 0.086 g) for ticks fed on control and 8-pSPT-treated mice, respectively (*t*-test: *t*_(8)_ = 2.343, *P* = 0.03, Fig. 2b). Analysis of the hatching rate and the larva survival period, revealed a reduction of 32% and 20% respectively, between the treated and untreated mice (Mann-Whitney U-test: U_(8)_ = 10, *P* = 0.004; Fig. 2d and Mann-Whitney U-test: U_(8)_ = 8, *P* = 0.01, Fig. 2e). The only parameter that was not changed between the groups was the capacity of female ticks to transform the ingested blood into eggs (Mann-Whitney U-test: U_(8)_ = 16, *P* = 0.10; i.e. reproductive index; Fig. 2c).

During the second infestation, the unique tick parameter that changed between the mice treated with the antagonist (8-pSPT) and the controls was the larva survival period, which was reduced by 12% on the antagonist-treated group (Table 3).

**Table 3:** Biological parameters analysed on tick-infested mice treated with 8-pSPT or saline

Groups	Engorged female weight (g) ± SEM	Egg mass weight (g) ± SEM	Reproductive index ± SEM	Larva hatching rate (%) ± SEM	Larva survival period (days) ± SEM
First infestation					
Saline	0.155 ± 0.01	0.084 ± 0.01	54.19 ± 1.67	88.56 ± 0.44	42.50 ± 0.59
8-pSPT	0.115 ± 0.01	0.058 ± 0.01	44.19 ± 6.46	66.63 ± 12.58	33.86 ± 3.07
*P*-value	0.04*	0.03*	0.16	0.01*	0.01*
Second infestation					
Saline	0.131 ± 0.01	0.078 ± 0.01	58.96 ± 2.08	83.57 ± 3.95	45.71 ± 1.59
8-pSPT	0.105 ± 0.01	0.055 ± 0.01	46.36 ± 7.35	77.13 ± 11.99	40.14 ± 1.66
*P-*value	0.18	0.12	0.14	0.24	0.03*

*P <0.05 comparing the 8-pSPT-treated group with the control group (saline)

*N* = 10/group; 8-pSPT, non-specific antagonist for ADO receptors

So far, our results suggest that the blockade of ADO receptors particularly affected the performance of ticks during the first infestation (Table 3).

### Deficiency of the A2a receptor in mice did not impair the biological and reproductive parameters of ticks during infestation

Tick-infested A2aR−/− mice did not present change in the number of engorged females, the weight of the engorged females, egg mass weight, reproductive index and larva hatching rate (Table 4).

**Table 4:** Biological parameters analysed on WT and A2aR−/− tick-infested mice

Groups	Engorged female (%) ± SEM	Engorged female weight (g) ± SEM	Egg mass weight (g) ± SEM	Reproductive index ± SEM	Larva hatching rate (%) ± SEM
First infestation					
WT	33 ± 7.02	0.105 ± 0.02	0.69 ± 0.01	56 ± 3.85	61 ± 45
A2aR−/−	53 ± 12.37	0.113 ± 0.01	0.69 ± 0.01	53 ± 2.86	88 ± 25
*P*-value	0.11	0.74	0.98	0.65	0.06

*N* = 10/group

### Treatment of tick-infested mice with an ADO receptors antagonist modulated DCs and altered lymph node cytokine production

To investigate whether treatment with 8-pSPT alters the immune response of mice infested with ticks, we performed one more infestation (third infestation) and collected the draining lymph nodes to determine the expression of stimulatory and co-stimulatory molecules CD40, CD86, CD80 and MHCII on DCs (CD11c^+^ cells) as also the production of cytokines.

**Fig. 3:**
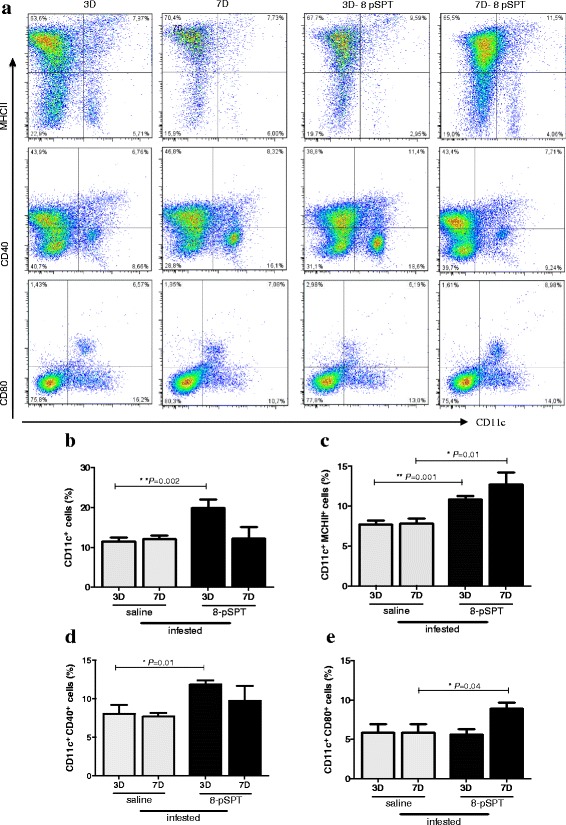
DCs activation after treatment with ADO receptor antagonist in mice infested with ticks. BALB/c mice (*n* = 6/group) were three times tick-infested and treated daily, throughout all infestations with a non-selective antagonist of ADO (8-pSPT, 20 mg/kg ip) or saline. Mice were killed on the 3rd and 7th day (3D and 7D) of the third infestation and their lymph nodes were harvested. Cells were labelled with the indicated antibody and analysed by flow cytometry. Data shown in **a**, **b**, **c**, **d** and **e** are presented as the mean percentage of CD11c + cells expressing CD80, CD40 or MHCII ± SEM from two independent experiments

Our data showed that on the third day of infestation, the 8-pSPT-treated group presented a significant increase in the percentage of DCs (CD11c^+^) (t-test: *t*_(8)_ = 3.576, *P* = 0.002) and CD11c^+^ CD40^+^ cells (t-test: *t*_(2.973)_ = 2.973, *P* = 0.01) compared to the controls, however the same was not detected on the seventh day (Fig. 3a-d). Furthermore, the treatment with 8-pSPT induced a significant increase in the percentage of CD11c^+^MHCII^+^ cells in the both days evaluated (Fig. 3c), while an enhanced frequency of CD11c^+^CD80^+^ cells was observed only on the seventh day (Fig. 3e). Concerning the percentage of CD11c^+^CD86^+^ cells, no significant differences between the 8-pSPT-treated or control group were observed (data not shown).

**Fig. 4:**
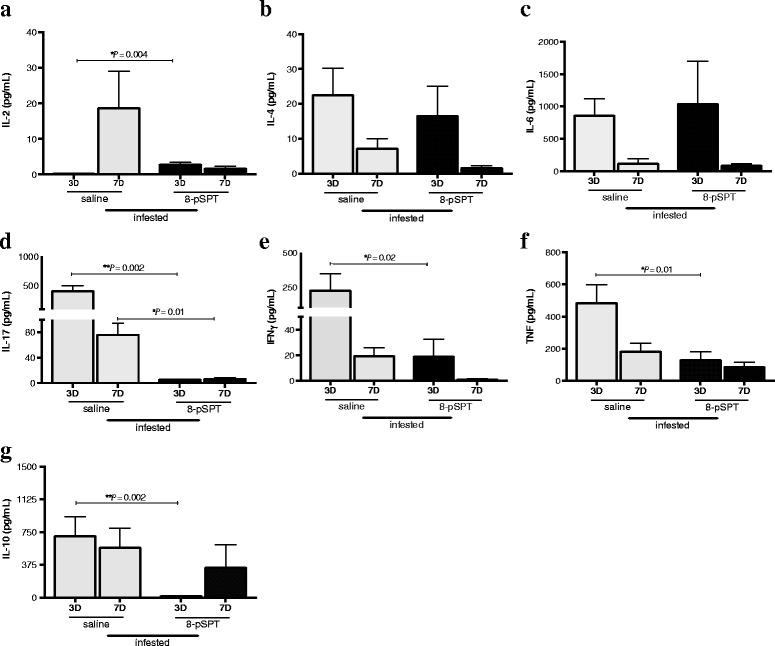
Cytokine production after treatment with ADO receptor antagonist in mice infested with ticks. BALB/c mice (6/group) were three times tick-infested and treated daily, throughout all infestations with a non-selective antagonist of ADO (8-pSPT, 20 mg/kg i.p.) or saline. Mice were killed on the 3rd and 7th day (3D and 7D) of the third infestation and their lymph nodes cells were cultured with Con-A (2 mg/ml). After 24 h of incubation, the supernatants were collected and assayed for IL-2 (**a**), IL-4 (**b**), IL-6 (**c**), Il-17 (**d**), INFγ (**e**), TNF (f) and IL-10 (**g**) cytokines by CBA. Data are presented as the mean of cytokine production ± SEM from two independent experiments

To investigate the effect of the 8-pSPT treatment on the cytokine production by cells of the lymph nodes of tick-infested mice, we stimulated the cell cultures with Con-A and the Th1, Th2 and Th17 cytokines profiles in the supernatant were measured by CBA (Fig. 4). On the third day of the third infestation, the production of IL-2 increased by 10 times for the cells of 8-pSPT-treated mice compared the control group (Mann-Whitney U-test: U_(6)_ = 1, *P* = 0.004) (Fig. 4a). Additionally, on the same third day, the treated group showed a significant decrease in the production of IL-17 (Mann-Whitney U-test: U_(6)_ = 0, *P* = 0.002), TNF-α (t-test: *t*_(6)_ = 2.810, *P* = 0.01), IFN-γ (Mann-Whitney U-test: U_(6)_ = 4, *P* = 0.02) and IL-10 compared to the controls (Mann-Whitney U-test: U_(6)_ = 0, *P* = 0.002) (Fig. 4d-g). No differences in the production of IL-4 and IL-6 were detected in the supernatants of cells from both groups (Fig. 4b, c).

## Discussion

It is known that the expression of ADO and PGE2 receptors is regulated during physiological and pathological stress [45] when both are upregulated to suppress cell activity. As previously described, *R. sanguineus* ticks produce high concentrations of ADO and PGE2, which accumulate in tick saliva and are inoculated into the host skin during feeding [29]. This was also reported for other tick species, such as *Amblyomma americanum* for ADO [46] and *Dermacentor variabilis* for PGE2 [47]. The latter showed that ticks use salivary PGE2 to impair macrophages to produce pro-inflammatory mediators and recruit fibroblasts to the feeding site, consequently inhibiting wound healing [47]*.* Therefore, a better understanding of the dynamic of expression of ADO and PGE2 receptors in the host skin during an infestation can help to unravel the role of these molecules in the success of the tick to achieve a good blood meal.

Here, we demonstrate that there is a significant increase in gene expression for the A2b and A3v1 receptors on the third day of a tick infestation on BALB/c mice. Previously, others have demonstrated that the A2b and A3 receptors are upregulated in inflammation, stress or injury and that blocking them with the use of selective antagonists can be used as a treatment for inflammatory diseases, such as Parkinson’s disease, ischemia and cancer [36, 48–52]. Our study also showed that on the seventh day of infestation, the A2b and A3v1 receptors expression returned to control values. This may have happened because of the large volume of tick saliva already released into the host skin, which contains a variety of other immunosuppressive molecules that can modulate the inflammation independently from the ADO signalling pathway [11, 13].

In regard to the expression of mRNA for PGE_2_ receptors (EP2, EP3 and EP4), these were unchanged during the tick infestation at the time points analysed (data not shown), while EP1 receptor was not detected in the skin of mice, regardless they were infested or not with ticks (data not shown). This latter observation corroborates other studies that show that, unlike other PGE2 receptors, the EP1 receptor is not expressed in the skin of mice [53].

Concerning that tick infestation modulates the expression of mRNA for A2b and A3v1 receptors in mice, we treated them daily with a non-selective antagonist of ADO receptors (8-pSPT) during the first and second infestation to evaluate the importance of ADO signalling pathway in the performance of ticks. Data indicate that the blockade of ADO receptors induces a significant reduction in the biological and reproductive parameters of ticks during the first infestation on mice, whereas during the second infestation this did not occur. A possible explanation can be that ticks produce different components within the time-course of feeding [54, 55]. Thus, additional regulatory molecules can be introduced into the host by the 7th day of infestation.

Although not having found a difference in the expression of A2aR on tick infested mice and knowing that ADO from *Phlebotomus papatasi* can suppress the immune response via A2aR [42], we tested if the lack of this receptor in mice (A2aR−/−) could reduce tick infestation performance. Our results indicated that this deficiency was not enough to compromise tick-feeding success, suggesting that other receptors (i.e. A2bR and A3v1R) are involved in the immunosuppression. This possibility has yet to be tested.

Our results indicate that ADO of tick saliva possibly plays an important role in suppressing host immune/inflammatory mechanisms, at least in the initial phase of tick feeding. This can happen, since other studies show that ADO can reduce the expression of adhesion molecules in endothelial cells, resulting in a decrease of leukocytes rolling, adhesion and migration to inflamed tissue [56, 57]. In addition, ADO can also increase vasodilation and may inhibit platelet aggregation [58, 59], modifications that may well assist ticks while they feed.

Tick saliva also impairs the differentiation and biological activity of DCs by reducing their stimulatory and co-stimulatory molecules in vitro*,* and this was associated to ADO [26–29] and other molecules, such as Salp 15 and Japanin [25]. Moreover, others have shown that the A2b receptor is the mediator of ADO effects on DCs, which results in impaired maturation (reduced expression of MHCII and CD86) and immunogenicity of DCs [35, 60]. Our data demonstrate that blocking ADO’s receptors (8-pSPT-treated mice) can increase the percentage of DCs, as also as their activation state (augmented expression of CD40, CD80 and MHCII). It seems that compromising the initial steps of the immune response to inhibit the differentiation and maturation of DCs into functional antigen-presenting cells can impair tick rejection by the host.

Besides being a non-selective blocker of adenosine receptors, 8-pSPT (theophylline) also presents a phosphodiesterase (PDE) inhibitor activity [61]. The effect of selective inhibition of PDE4 (the main PDE expressed in immune cells) includes reduced secretion of IL-12 and TNF-α by DCs and impaired capacity to promote Th1-polarized responses [62, 63]. Impaired secretion of IL-12 and TNF-α by DC was observed in our work after 8-pSPT treatment, what could suggest some influence of PDE inhibition, even though theophylline is considered a weak inhibitor of PDE4, compared with its inhibitory activity on A1 and A2 ADO receptors [64]. However, we also observed a decreased production of IL-10 by DCs, contrasting with the already described enhancement of IL-10 secretion by immune cells when PDE4 is inhibited [65]. Indeed, the increase of IL-10 is seen when DCs are stimulated with the non-selective ADO agonist NECA [35] and during tick infestations [66], while the treatment of DCs with a selective antagonist of the A2B receptor (which is upregulated during tick infestation, Fig. 4) impairs IL10 production [35]. In addition, PDE4 selective inhibition did not promote up-regulation of surface expression of HLA-DR, CD40 and CD80 like was observed when DCs were cultured in the presence of a non-selective agonist of ADO receptors NECA [35], corroborating our reasoning. Broadly, the DC phenotype promoted by PDE4 inhibition is similar to the phenotype presented during tick saliva’s stimulus and is related with mice susceptibility to ticks, and not resistance, as shown in our work, what strongly suggests that our results are more to be due to ADO signalling than PDE signalling.

We have previously shown that ADO from tick saliva modulates cytokine production, downregulating IL-12p40 and TNF-α, and upregulating IL-10 production by DCs [29]. In line with this report, it has been shown that ADO from human and mice impairs TNF-α, IL-6, and IL-12 production, while it augments secretion of IL-10 by DCs and macrophages [67–69]. Here we have shown that the ADO receptors blockade can revert the modulation by tick saliva. In this way, tick-infested mice treated with 8-pSPT induced a mixed cytokine profile, with a decrease of TNF, IFN-γ, IL-10 and IL-17 and increased of IL-2 production. The reduction of IL-17 is in line with other studies that show that, in autoimmune diseases models, the blockade of A2b receptors diminishes Th17 type cytokines [70]. On the other hand, the A2b receptor has also been shown to modulate the IL-6 production [71], a phenomenon that was not seen in this study.

Others have shown that ADO, per se, also inhibits T cell proliferation and reduces the synthesis of IL-2 [36, 56, 72, 73], while can increase the numbers of T regulatory cells through an increase in IL-10 at the inflamed site, which can inhibit the activity of effector T cells [74]. Moreover, the inhibition of T cell proliferation by ADO has been shown to occur via activation of the A2b receptor [75, 76]. Related to the role of A3 receptors in inflammation there is still a controversial debate. Some studies found that A3 receptor activation can improve inflammatory mediators release and degranulation in mast cells, suggesting a pro-inflammatory role [77, 78]. Other works have shown that its activation can suppress TNF-α release from LPS-stimulated macrophages and superoxide production and chemotaxis of neutrophils besides its competence to suppress the proliferation T cells [34, 78–81]. Interestingly, a recent study indicates a common association of the A3 receptor with potent analgesic effect in rodent models of chronic pain [82]. Maybe tick saliva’s ADO signs via A3 receptor in the early phase of feeding, so it can get its blood meal properly by blocking pain.

## Conclusion

Taken together, the results demonstrate that ADO, previously identified in the saliva of ticks, can bind to A2b and A3 receptors on host cells. As doing so, it inhibits the function of diverse cells of the host immune response, including dendritic and T cells, modulating both the innate immune response and the acquired immune response, inducing a mixed T cell type response that facilitates tick-feeding and reproduction.

## Abbreviations

8-pSPT: 8-(p-Sulfophenyl)theophylline
A2aR−/−: Deficiency of the A2a receptor
ADO: Adenosine
Con-A: Concanavalin A
DCs: Dendritic cells
I-A/I-E: MHCII
KO: Knockout
PGE2: Prostaglandin E2
WT: Wild-type
